# Characterization of the complete mitochondrial genome of the medical fungus *Ganoderma resinaceum* Boud., 1889 (Polyporales: *Ganoderma*taceae)

**DOI:** 10.1080/23802359.2024.2410449

**Published:** 2024-09-30

**Authors:** Mingda He, Guangjiu Chen

**Affiliations:** a Chengdu Sport University, Chengdu, P. R. China; b Luzhou Vocational and Technical College, Luzhou, P. R. China

**Keywords:** Mitochondrial genome, *Ganoderma*, evolution, phylogeny, medical mushroom

## Abstract

The medical mushroom *Ganoderma resinaceum* Boud., 1889, is of great interest in pharmacy due to its diverse functional active ingredients. However, the mitochondrial genome of *G. resinaceum* remains unexplored. Here, we present the complete mitochondrial genome of *G. resinaceum*, which spans 67,458 bp and has a GC content of 25.65%. This genome encompasses 15 core protein-coding genes, 8 independent ORFs, 15 intronic ORFs, 27 tRNAs, and 2 rRNA genes. Through phylogenetic analysis using Bayesian inference (BI), we elucidated the evolutionary relationships among 34 Basidiomycota fungi, revealing distinct clades and indicating a close relationship between *G. resinaceum* and *G. subamboinense*.

## 1. Introduction

*Ganoderma resinaceum* Boud., 1889, a species of the renowned *Ganoderma* genus, has attracted the attention of researchers and traditional medicine practitioners alike for its exceptional therapeutic properties (Chen et al. 2017, 2022; Buratti et al. 2023). This medicinal fungus has been used for centuries to promote health, enhance immunity, and treat various ailments (Peng et al. 2013; Kou et al. 2022). Modern research has revealed that *G. resinaceum* contains a rich array of bioactive compounds, including polysaccharides, triterpenoids, and sterols, which are responsible for its diverse pharmacological effects (Chen et al. 2018; Huang et al. 2020; Gauna et al. 2021). These compounds exhibit immunomodulatory, anti-inflammatory, and antitumor properties, making *G. resinaceum* a promising candidate for drug development (Yang et al. 2020; Sipping et al. 2022).

The mitochondrial genome of eukaryotic organisms is crucial for regulating growth, development, cellular homeostasis, and responses to environmental stimuli (Ernster and Schatz 1981; McBride et al. 2006; Murphy 2009). It has been proposed that the mitochondrial genome is a useful resource for studying fungal phylogeny (Xu and Wang 2015; Li, Bao, et al. 2022; Li, Li, et al. 2022; Li, Luo, et al. 2023). Due to the abundance of homonyms and homonyms in the cultivation of *Ganoderma* species, the systematic development and classification of the *Ganoderma lucidum* complex are challenging tasks (Zhou et al. 2015). Up to now, over 190 species are taxonomically accepted in *Ganoderma*, making it as one of the most species-rich genera in Ganodermataceae (He et al. 2022, 2024). The mitochondrial genome characteristics of fungi within the *Ganoderma* genus have been insufficiently explored, with only 12 mitochondrial genomes of *Ganoderma* species reported to date (Woo et al. 2003; Joardar et al. 2012; Li et al. 2017; Wu et al. 2023). This study revealed the primary complete mitochondrial genome of *G. resinaceum*, enhancing our understanding of the genomic traits of this crucial fungal group.

## 2. Materials and methods

### 2.1. Sample collection

**Figure 1.**
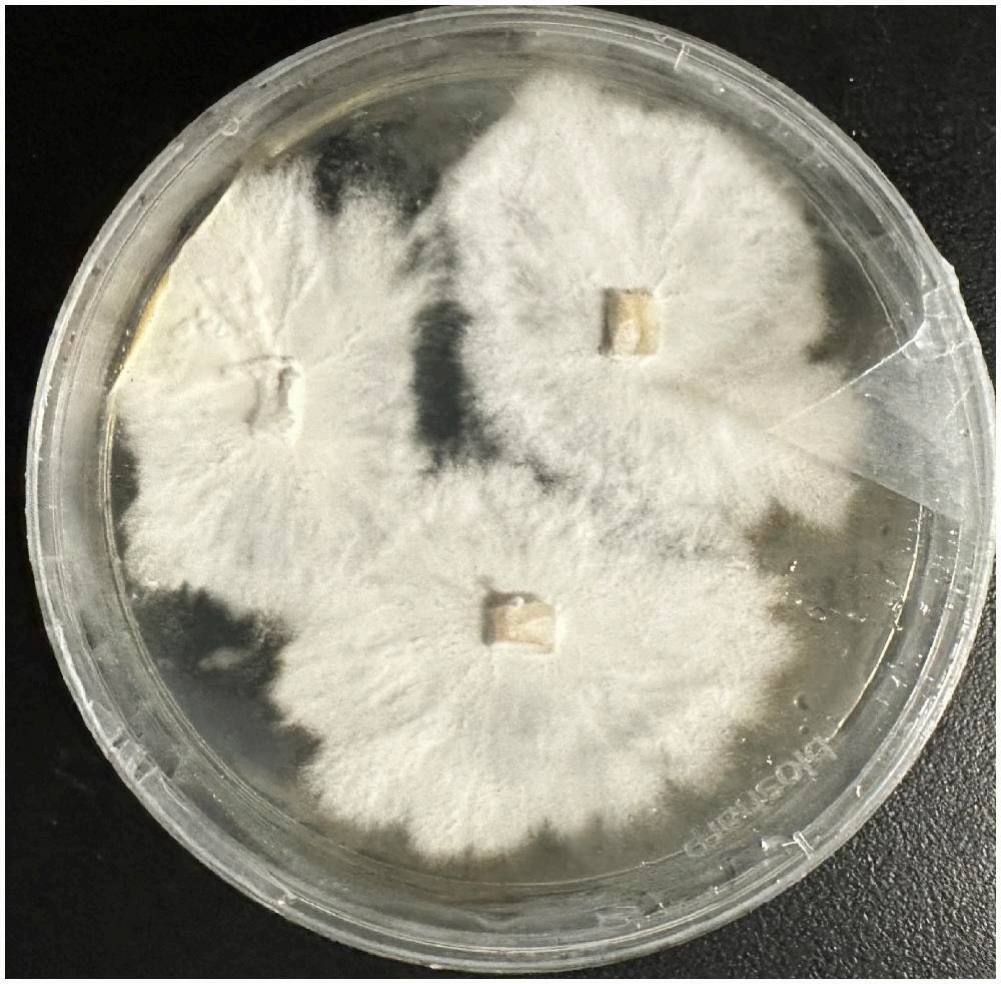
The microscopic hyphal morphology of *Ganoderma resinaceum*. A photo of the species was taken by Guangjiu Chen.

A specimen of *G. resinaceum* was obtained from Chengdu, Sichuan, China (105.55°E, 30.69°N) in 2023. Identification was based on morphological examination and ITS rDNA sequencing (Du et al. 2019; Tchotet Tchoumi et al. 2019). The specimens were deposited in the Culture Collection Center of Chengdu University under voucher number GS14. For further details, we kindly obtained Guangjiu Chen at 330115925@qq.com (Figure 1).

### 2.2. Mitochondrial genome assembly and annotation

A fungal DNA extraction kit from Omega Bio-Tek (Norcross, GA) was used to extract DNA from *G. resinaceum*, and sequencing library preparation was carried out using the NEBNext^®^ Ultra^™^ II DNA Library Prep Kit (NEB, Beijing, China) following the manufacturer’s guidelines. Whole-genome sequencing was conducted on the Illumina HiSeq 2500 Platform (Illumina, San Diego, CA), followed by filtration of low-quality sequences using ngsShoRT (Chen et al. 2014) and elimination of adapter reads with AdapterRemoval v2 (Schubert et al. 2016) to ensure data precision. The mitochondrial genome of *G. resinaceum* was *de novo* assembled utilizing NOVOPlasty version 4.3.3 with a k-mer size of 29 (Dierckxsens et al. 2017). Annotation of the mitochondrial genome was carried out in accordance with established protocols (Li et al., 2019; Li, Ren, et al., 2020; Li, Xiao, et al., 2023) involving the use of the MFannot tool (Valach et al. 2014) and MITOS (Bernt et al. 2013). The NCBI Open Reading Frame Finder facilitates the prediction or modification of protein-coding genes (PCGs) or open reading frames (ORFs) that are longer than 100 amino acids (NCBI Resource Coordinators 2017). Protein-coding genes (PCGs) or open reading frames (ORFs) were annotated *via* BLASTP searches against the NCBI nonredundant protein sequence database (Bleasby and Wootton 1990). The use of exonerate version 2.2 aided in accurately identifying exon and intron boundaries in protein-coding genes (Slater and Birney 2005). The identification and validation of tRNA genes in the mitochondrial genome of *G. resinaceum* were accomplished using tRNAscan-SE v1.3.1 (Lowe and Chan 2016). The PMGmap online web tool (http://www.1 kmpg.cn/pmgmap) was utilized for visualizing intron-containing gene structures of the mitochondrial genome (Zhang et al. 2024).

### 2.3. Phylogenetic analysis

The phylogenetic tree was constructed using established methods commonly utilized for inferring mitochondrial genomic phylogeny (Li et al. 2021; Li, He, et al. 2020; Li, Zhang, et al. 2022). The alignment of individual mitochondrial genes (excluding intron regions) was conducted utilizing MAFFT v7.037 software (Katoh et al. 2019). The aligned mitochondrial genes were merged using SequenceMatrix v1.7.8, thereby producing a cohesive mitochondrial dataset (Vaidya et al. 2011). To determine potential phylogenetic variations among diverse mitochondrial genes, an initial partition homogeneity test was performed using PAUP v 4.0b10 (Swofford 2002), following established literature (Xiang et al. 2013). PartitionFinder 2.1.1 was utilized to determine the most suitable partitioning strategies and evolutionary models for the amalgamated mitochondrial dataset (Lanfear et al. 2017). The construction of phylogenetic trees using the Bayesian inference method was carried out with the software MrBayes v3.2.6 (Ronquist et al. 2012).

## 3. Results

**Figure 2.**
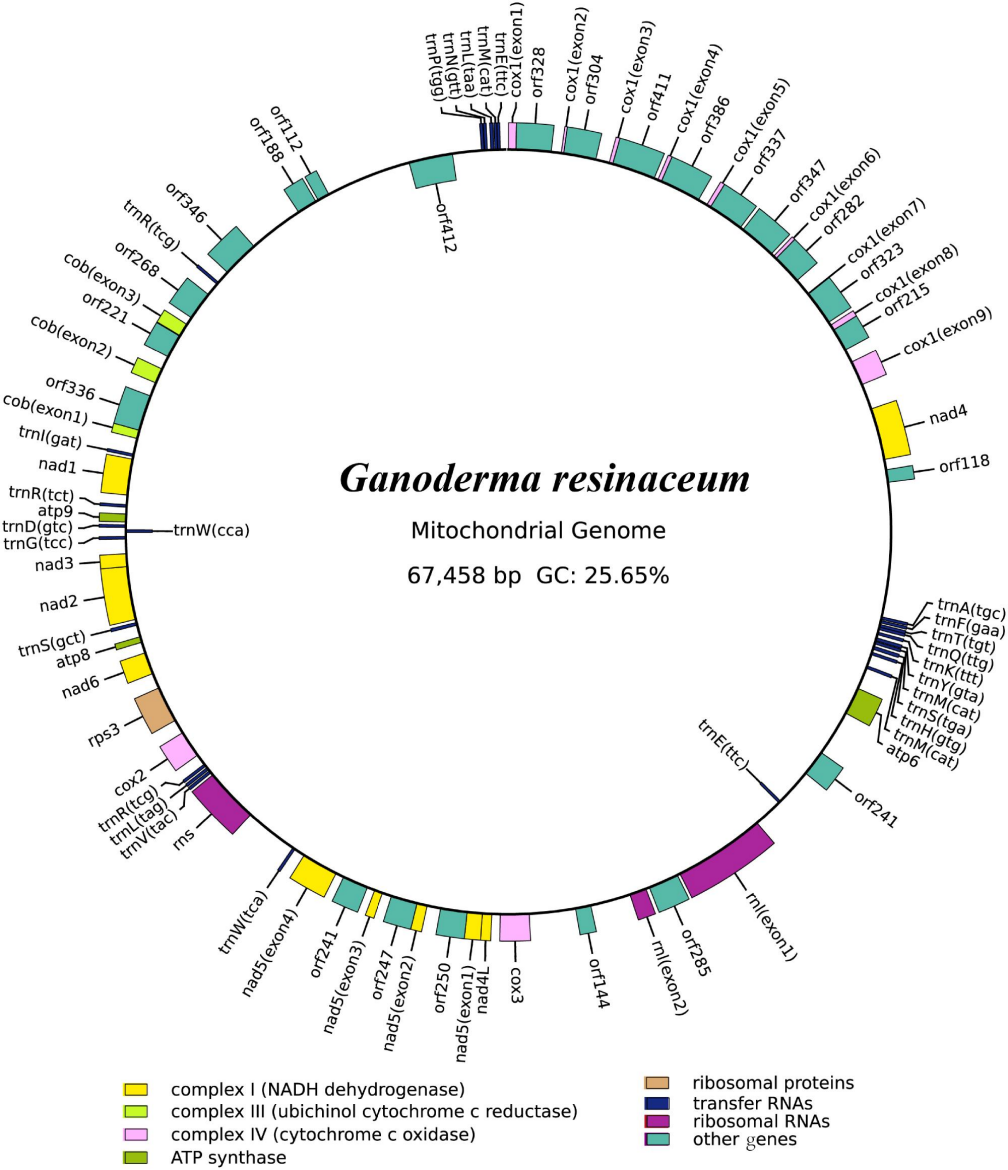
Circular mitochondrial genome map of *Ganoderma resinaceum*. Different color blocks represent different genes. Genes outside the loop indicate genes in the direct strand, while genes inside the loop indicate genes in the reverse strand.

**Figure 3.**
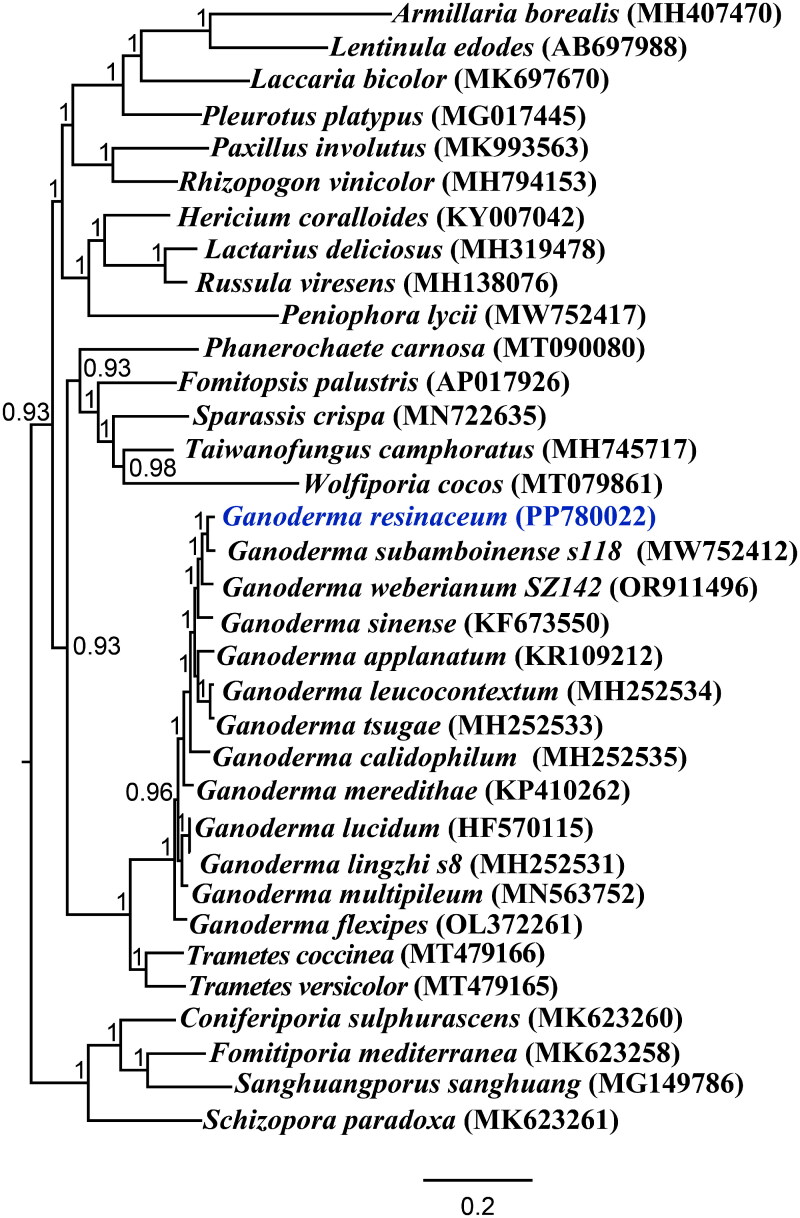
Bayesian inference (BI) tree generated using 14 concatenated mitochondrial protein-coding genes (*atp6*, *atp8*, *atp9, cob, cox1, cox2, cox3, nad1, nad2, nad3, nad4, nad4L, nad5,* and *nad6*) from *Ganoderma resinaceum* and 33 other Basidiomycota species. The accession numbers of the sequences were as follows: *Ganoderma meredithae* (KP410262) (Wang et al. 2016), *Ganoderma multipileum* (MN563752), *Ganoderma flexipes* (OL372261), *Ganoderma weberianum* SZ142 (OR911496), *Ganoderma lingzhi* s8 (MH252531) (Li et al., 2019), *Ganoderma subamboinense* s118 (MW752412) (Li et al. 2022), *Ganoderma calidophilum* (MH252535) (Li et al., 2019), *Ganoderma sinense* (KF673550), *Ganoderma leucocontextum* (MH252534) (Li et al., 2019), *Ganoderma lucidum* (HF570115) (Li et al. 2013), *Ganoderma applanatum* (KR109212) (Wang et al. 2016), *Ganoderma tsugae* (MH2533) (Li, Xiang, et al., 2019), *Peniophora lycii* (MW752417) (Gou et al. 2021), *Trametes coccinea* (MT479166) (Chen et al. 2021), *Trametes versicolor* (MT479165) (Chen et al. 2021), *Schizopora paradoxa* (MK623261) (Min et al. 2015), *Sanghuangporus sanghuang* (MG149786) (Han et al. 2018), *Fomitiporia mediterranea* (MK623258) (Lee et al. 2019), *Coniferiporia sulphurascens* (MK623260) (Lee et al. 2019), *Phanerochaete carnosa* (MT090080) (Wang et al. 2020), *Fomitopsis palustris* (AP017926), *Rhizopogon vinicolor* (MH794153) (Li, Ren, et al., 2019), *Paxillus involutus* (MK993563) (Li, Ren, et al. 2020), *Armillaria borealis* (MH407470) (Kolesnikova et al. 2019), *Pleurotus platypus* (MG017445) (Li, Chen, et al. 2018), *Lentinula edodes* (AB697988), *Laccaria bicolor* (MK697670) (Li, Yang, et al. 2020), *Taiwanofungus camphoratus* (MH745717) (Wang et al. 2020), *Sparassis crispa* (MN722635), *Lactarius deliciosus* (MH319478) (Li, Wang, et al., 2019), *Hericium coralloides* (KY007042) (Zhang et al. 2017), *Russula viresens* (MH138076) (Li, Wang, et al. 2018), and *Wolfiporia cocos* (MT079861) (Chen et al. 2020).

The average depth of the coverage-depth map was 6336.92×, as shown in Supplementary Figure 1. The mitochondrial genome of *G. resinaceum* is 67,458 bp in length, with a GC content of 25.65%. The gene structures that include introns are depicted in Supplementary Figure 2. The mitochondrial genome of *G. resinaceum* consists of 37.11% adenine, 13.49% guanine, 37.24% thymine, and 12.16% cytosine. Examination of the mitochondrial genome of *G. resinaceum* revealed 38 open-reading frames, including 15 core protein-coding genes (*cox1*, *cox2*, *cox3*, *atp6*, *atp8*, *atp9*, *cob*, *nad1*, *nad2*, *nad3*, *nad4*, *nad4L*, *nad5*, *nad6*, and *rps3*), 8 independent ORFs, and 15 intronic ORFs, as illustrated in Figure 2. Notably, the functions of proteins encoded by free-standing ORFs remain unknown. The mitochondrial genome of *G. resinaceum* contains 14 introns, comprising 10 Group IB, 2 Group ID, 1 Group IA intron, and 1 with unknown types. The *cox1* gene harbored 8 introns, which encoded 9 intronic ORFs, including 7 LAGLIDADG endonuclease and 2 GIY endonuclease. The *rnl* gene harbored 1 intron, which encoded LAGLIDADG endonuclease. The *nad5* gene harbored 3 introns, all of which encoded LAGLIDADG endonuclease. In addition, the *cob* gene harbored 2 introns, one of which encoded LAGLIDADG endonuclease, and the other encoded GIY endonuclease. Certain introns harbor intronic open reading frames (ORFs) that encode LAGLIDADG-homing endonucleases or GIY-YIG-homing endonucleases. In addition, the mitochondrial genome of *G. resinaceum* includes two ribosomal RNA genes, the small subunit (*rns*) and the large subunit (*rnl*), as well as 27 transferred RNA genes. Phylogenetic analysis based on the GTR+I + G model revealed that *G. resinaceum* was most closely related to *G. subamboinense* (Li et al. 2022), as depicted in Figure 3.

## 4. Discussion and conclusion

The utilization of the mitochondrial genome enhances the understanding of phylogenetic relationships among species (Zhang et al. 2020, 2022, 2023; Ren et al. 2021; Gao et al. 2024). The mitochondrial genome can provide rich genetic information for revealing the dynamic changes in mitochondrial sequence and structure in eukaryotes, and analyzing the phylogenetic relationships between species (Li, Xie, et al. 2023; Feng et al. 2024). The unavailability of a mitochondrial reference genome for *G. resinaceum* restricts the application of mitochondrial genomes for classifying and examining the phylogenetic relationships within *Ganoderma* fungi (Li, Xiang, et al., 2019; Li, Zhang, et al., 2022; Meng et al. 2022). In this study, the entire mitochondrial genome of a *Ganoderma* species was sequenced, revealing a length of 67,458 bp and a GC content of 25.65%. The genome consists of 15 core protein-coding genes (PCGs), 8 autonomous ORFs, 15 intronic ORFs, 27 tRNAs, and 2 rRNA genes. Compared with those of the remaining 16 *Ganoderma* strains, the mitochondrial genome of *G. resinaceum* is moderate in size (Li et al., 2019). In the phylogenetic analysis conducted using the Bayesian inference (BI) method, *G. resinaceum* was positioned in closest proximity to *G. subamboinense* within a group of 34 Basidiomycota species (Li et al. 2022), with strong support for the main clades. This research improves our understanding of *Ganoderma* species differentiation, mitochondrial evolution, and diversity within this crucial fungal group (Zhou et al. 2015; Du et al. 2019; He et al. 2022; Mardones et al. 2023).

## Supplementary Material

Supplementary figure 2.docx

## Data Availability

The genome sequence data that support the findings of this study are openly available in the NCBI GenBank at https://www.ncbi.nlm.nih.gov/under accession no. PP780022. The associated BioProject, SRA, and Bio-Sample numbers are PRJNA1110369, SRR28997063 and SAMN41345065, respectively.
