# Supplementary material for: Characterization of the complete mitochondrial genome of the medical fungus Ganoderma resinaceum Boud., 1889 (Polyporales: Ganodermataceae)
Source: Mitochondrial DNA B Resour. 2024 Sep 30;9(10):1291–7. doi: 10.1080/23802359.2024.2410449 (PMC11445931; doi:10.1080/23802359.2024.2410449)
Supplement: Supplementary figure 2.docx [file TMDN_A_2410449_SM3105.docx]

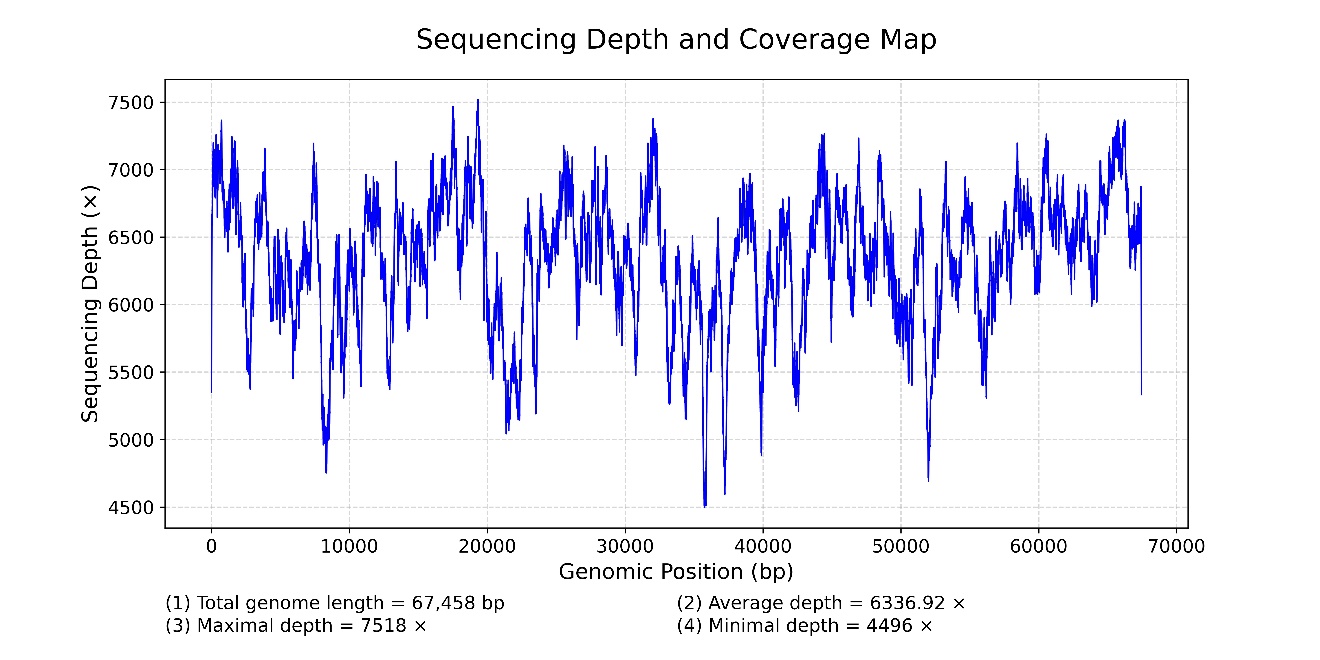


**Figure S1** Sequencing depth and coverage map of *Ganoderma resinaceum* mitochondrial genome.


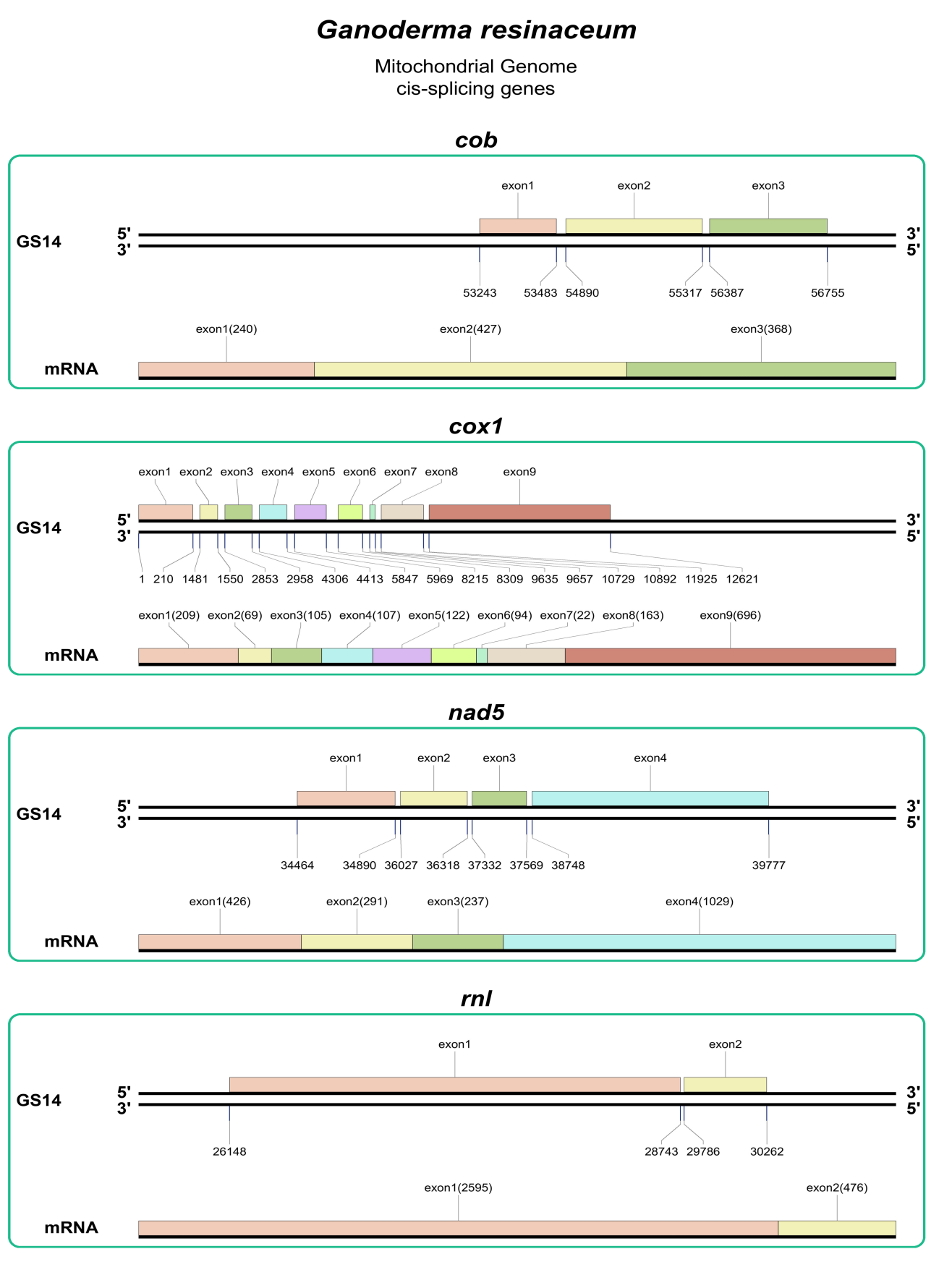


**Supplementary Figure S2** Cis-splicing genes of the *Ganoderma resinaceum* mitochondrial genome.
